# Chronic Exposure to High Altitude: Synaptic, Astroglial and Memory Changes

**DOI:** 10.1038/s41598-019-52563-1

**Published:** 2019-11-11

**Authors:** Rupali Sharma, Nathan P. Cramer, Bayley Perry, Zahra Adahman, Erin K. Murphy, Xiufen Xu, Bernard J. Dardzinski, Zygmunt Galdzicki, Daniel P. Perl, Dara L. Dickstein, Diego Iacono

**Affiliations:** 1 Department of Pathology, F. Edward Hébert School of Medicine Uniformed Services University of the Health Sciences (USU), Bethesda, MD USA; 2 Center for Neuroscience and Regenerative Medicine (CNRM), F. Edward Hébert School of Medicine, Uniformed Services University of the Health Sciences (USU), Bethesda, MD USA; 3 Department of Anatomy, Physiology and Genetics, F. Edward Hébert School of Medicine, Uniformed Services University of the Health Sciences (USU), Bethesda, MD USA; 4 Department of Radiology and Radiological Sciences, F. Edward Hébert School of Medicine, Uniformed Services University of the Health Sciences (USU), Bethesda, MD USA; 5 Department of Neurology, F. Edward Hébert School of Medicine, Uniformed Services University of the Health Sciences (USU), Bethesda, MD USA; 6 Neurodegenerative Clinics, National Institute of Neurological Disorders and Stroke (NINDS), NIH, Bethesda, MD USA; 7 The Henry M. Jackson Foundation for the Advancement of Military Medicine (HJF), Bethesda, MD USA

**Keywords:** Neuroscience, Outcomes research

## Abstract

Long-term operations carried out at high altitude (HA) by military personnel, pilots, and astronauts may trigger health complications. In particular, chronic exposure to high altitude (CEHA) has been associated with deficits in cognitive function. In this study, we found that mice exposed to chronic HA (5000 m for 12 weeks) exhibited deficits in learning and memory associated with hippocampal function and were linked with changes in the expression of synaptic proteins across various regions of the brain. Specifically, we found decreased levels of synaptophysin (SYP) (*p* < 0.05) and spinophilin (SPH) (*p* < 0.05) in the olfactory cortex, post synaptic density−95 (PSD-95) (*p* < 0.05), growth associated protein 43 (GAP43) (*p* < 0.05), glial fibrillary acidic protein (GFAP) (*p* < 0.05) in the cerebellum, and SYP (*p* < 0.05) and PSD-95 (*p* < 0.05) in the brainstem. Ultrastructural analyses of synaptic density and morphology in the hippocampus did not reveal any differences in CEHA mice compared to SL mice. Our data are novel and suggest that CEHA exposure leads to cognitive impairment in conjunction with neuroanatomically-based molecular changes in synaptic protein levels and astroglial cell marker in a region specific manner. We hypothesize that these new findings are part of highly complex molecular and neuroplasticity mechanisms underlying neuroadaptation response that occurs in brains when chronically exposed to HA.

## Introduction

Exposure to high altitude (HA) can have significant effects on brain function^1,2^. HA can range from 1500–3500 meters (m) (~4,900–11,500 feet [ft]) (HA); 3500–5500 m (~11,500–18000 ft) (very HA), and above 5500 m (>18,000 ft) (extreme HA) in comparison to sea level (SL)^3,4^. People who work, travel or are trained at HA (i.e. military service members during deployment periods, miners, astronomers, and astronauts) can encounter phenomena of hypobaric hypoxia (HH) that may generate major consequences in terms of adaptation in various organs such as lungs, heart, circulatory system, and the central nervous system (CNS)^5^. Specifically, sudden ascent and chronic exposure to HA (CEHA) can be associated with a series of neurological effects including headache^6,7^, loss of appetite, nausea^8,9^, sleep patterns, changes in mood, and deficits of cognitive functions^10^. In the military, HA has been shown to have an impact on the CNS and respiratory system generating neurological and pulmonary syndromes with longer term consequences in mental efficiency, military readiness and operational capabilities^11^. While some of these HA-associated neurological problems can be potentially reduced by gradual periods of altitude acclimatization, there are operational circumstances where gradual periods of acclimatization are not possible, such as immediate military actions requiring unexpected periods of CEHA.

In general, while acute exposure to high altitude (AEHA) and related clinical syndromes, such as cerebral venous thrombosis, seizures, transient ischemic attacks, pulmonary and cerebral edema are relatively known^12^, the effects of CEHA are not fully elucidated, yet. It has been shown that CEHA and its consequent hypoxic/hypobaric effects are capable of inducing changes in motor behavior along with reduction in cognitive performance due to maladaptive changes of the CNS^13–18^. Animal studies have shown that CEHA is capable of inducing changes in motor and cognitive behavior suggesting that the brain continually adapts to the stress caused by CEHA^19,20^. In Mount Everest climbers, cognitive and linguistic performance were assessed at different time points, and it was found that there were more errors in speech at increased altitudes, which, itself, is an indication of cognitive deficit reflecting degraded cortical and basal ganglia activities^14^. Moreover, a recent report on U-2 pilots operating at 29,000 ft have shown a lower performance in cognitive tests associated with white matter abnormalities^17,18^.

It is well known that the CNS, and in particular neurons, are susceptible to the deleterious effects of diminished levels of oxygen and glucose, which are some of the main atmospheric and metabolic factors associated with changes as a result of exposure to HA^21^. Whether CEHA, kept at sub-infarction levels, leads to specific molecular changes in the brain has not been explored. To investigate the underlying molecular and related neuroadaptive effects of CEHA, we utilized our well-established and previously characterized mouse model of HA where animals are exposed to chronic HA (5000 m) for 12 weeks^13,19,21^ and compared to mice kept at sea level (SL). Using this model we measured behavioral performance, synaptic morphological changes, and biochemical changes of different synaptic, astroglial and myelin proteins across different regions of the CNS (olfactory cortex, hippocampus, cerebellum and brainstem).

### Statistical analysis

Independent sample *t*-tests were used for all analyses. The α level was set at 0.05 with values of *p* < 0.05 considered statistically significant. All statistical analyses were carried out using Prism 7.00 software (GraphPad software, La Jolla, CA USA).

## Results

### CEHA induces memory deficits

**Figure 1:**
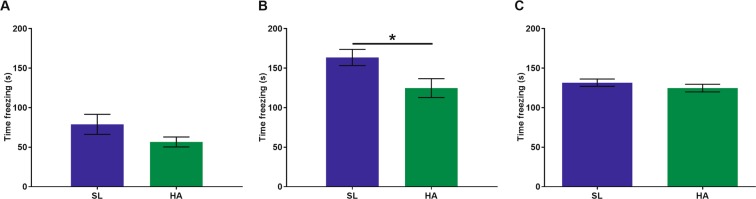
CEHA (12 weeks) results in hippocampal memory impairment. (**A**) No significant differences were observed during training. (**B**) HA mice spent significantly less time freezing when returned to the context test after 24hrs. (**C**) No significant differences were observed in the cued test. Data are expressed as mean ± SEM, n = 18 animals per group, *p < 0.05.

Exposure to HA has been shown to cause cognitive deficits^14,19,20,22,23^. We used the hippocampal fear conditioning paradigm to assess cognitive function in CEHA mice compared to SL controls. We found that during the training session, there was no significant difference in the amount of time HA mice spent freezing compared to SL mice (Fig. 1A). However, when HA mice returned to the training context 24 hours later, they spent significantly less time freezing compared to the SL mice (t = 2.463, *p* = 0.019), (Fig. 1B). No differences were observed between freezing behaviors in HA vs. SL mice during the cued tone (Fig. 1C). These results indicate that HA mice experienced significant learning deficits compared to SL mice.

### CEHA has no effects on synaptic density and spine morphology in the CA1 *striatum radiatum* of the hippocampus

**Figure 2:**
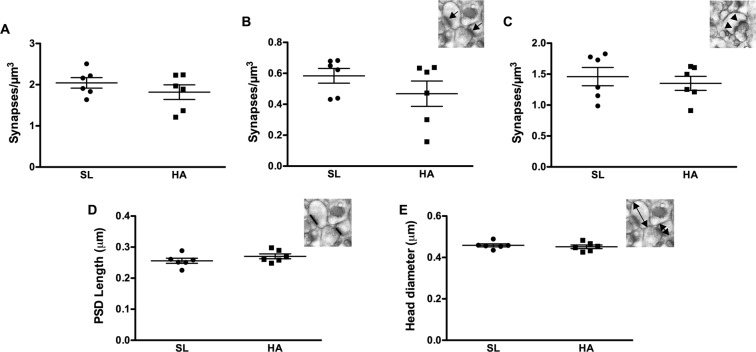
CEHA has no significant effect on synaptic density, PSD length or spine head diameter in the CA1 *striatum radiatum* of the hippocampus. (**A**) Total synapse density, (**B**) non-perforated synapse density (inset: arrows indicate single synapses), (**C**) perforated synapse density (inset: arrows indicate perforated-synapses), (**D**) PSD length (inset: black line indicate PSD), and (**E**) spine head diameter (inset: arrows indicate measured HD). Data represent group means ± SEM, n = 6 animals per group.

Having found that CEHA causes deficits in hippocampal memory, we initially examined the impact of CEHA on synaptic density and synaptic morphology in the *stratum radiatum* dendritic domain of the CA1 region of the hippocampus. Approximately 16,743 synapses were assessed (1,395 synapses per animal on average) across serial EM sections using the dissector method^24–27^. Analysis of total synaptic density revealed no significant differences between HA and SL mice (Fig. 2A). While we did not observe changes in synaptic density, it is still possible that there may be alterations in the types of synapses and morphology. Consequently, further analyses of the different synaptic types such as perforated and non-perforated, as well as post synaptic density (PSD) length and spine head diameter, revealed no significant differences between HA and SL mice (Fig. 2B–E).

### CEHA leads to decreased levels of synaptic and astroglial proteins across different anatomical regions of the brain

#### Olfactory cortex

**Figure 3:**
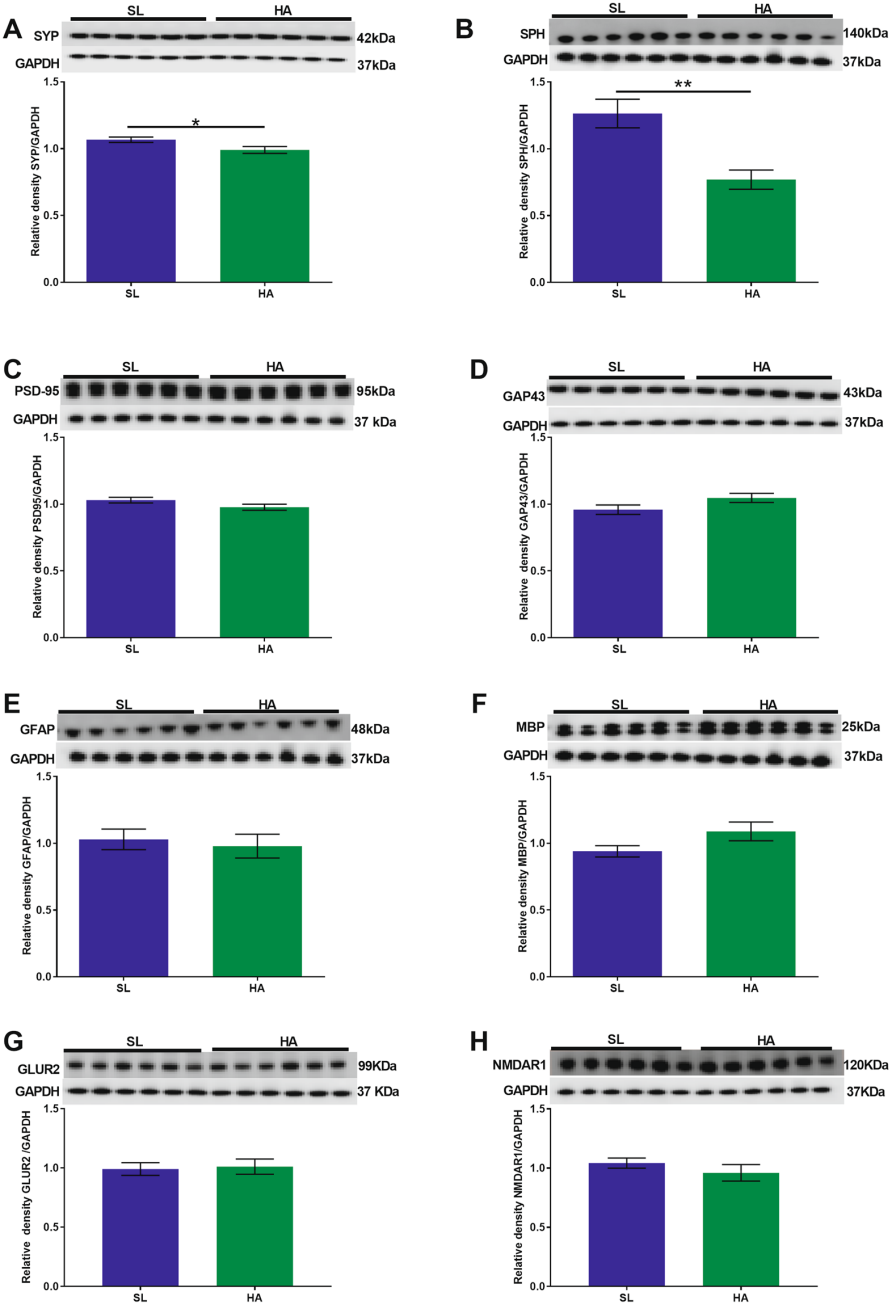
CEHA results in a significant decrease in the expression levels of SYP and SPH in the olfactory cortex. WB analysis of (**A**) SYP, (**B**) SPH, (**C**) PSD-95, (**D**) GAP43, (**E**) GFAP, (**F**) MBP (**G**) GLUR2, and (**H**) NMDAR1 protein levels. No significant changes were observed in PSD-95, GAP43, GFAP, MBP, GLUR2 and NMDAR1 protein levels. Data are expressed as mean ± SEM, n = 6 animals per group. *p < 0.05, **p < 0.01. Full length blots are presented in Supplementary Fig. 1 (GAPDH was done on each blot after stripping the protein of interest on each corresponding blot).

We observed decreased expression levels of SYP (t = 2.320; *p* = 0.042, Fig. 3A) and SPH (t = 3.838; *p* = 0.003, Fig. 3B) in HA vs. SL mice. No significant changes were observed in the expression levels of PSD-95, GAP43, GFAP, MBP (Fig. 3C–F), GLUR2 (Fig. 3G), and NMDAR1 (Fig. 3H) in HA mice compared to SL mice.

#### Hippocampus

**Figure 4:**
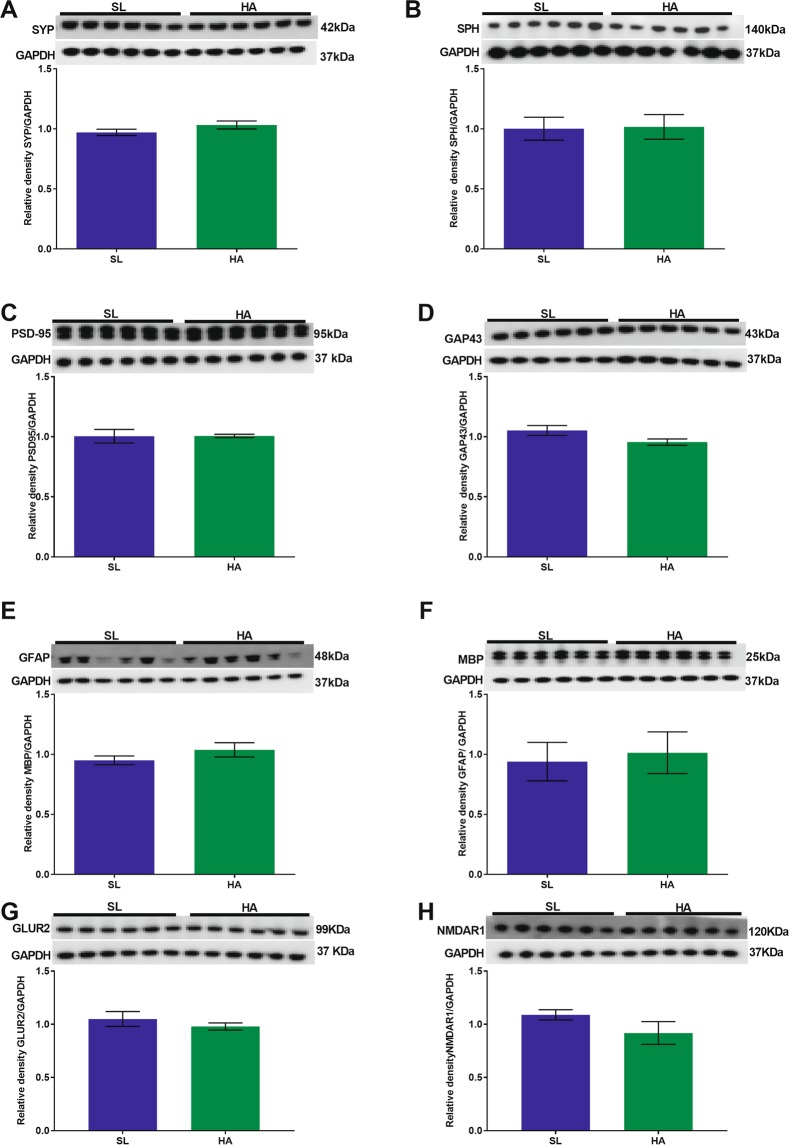
CEHA does not cause significant changes in synaptic protein expression levels in the hippocampus. WB analysis of (**A**) SYP, (**B**) SPH, (**C**) PSD-95, (**D**) GAP43, (**E**) GFAP, (**F**) MBP, (**G**) GLUR2, and (**H**) NMDAR1 protein levels. Data are expressed as mean ± SEM, n = 6. Full length blots are presented in Supplementary Fig. 2 (GAPDH was done on each blot after stripping the protein of interest on each corresponding blot).

There were no significant differences in the expression levels of SYP, SPH, PSD-95, GAP43, GFAP, MBP (Fig. 4A–F), GLUR2 (Fig. 4G) and NMDAR1 (Fig. 4H) in HA mice compared to SL mice.

#### Cerebellum

**Figure 5:**
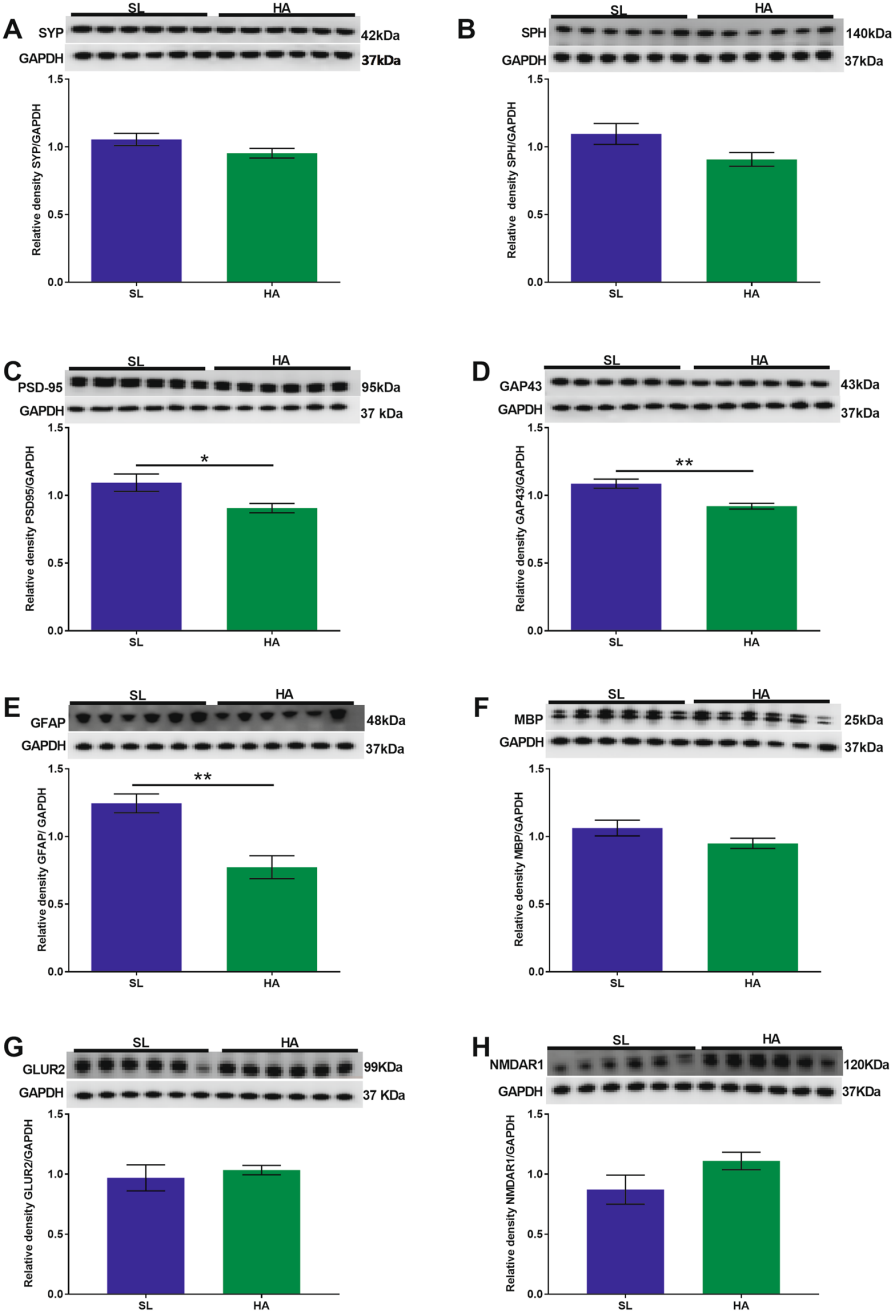
CEHA results in a significant decrease in the expression levels of PSD-95, GAP43, and GFAP in the cerebellum. WB analysis of (**A**) SYP, (**B**) SPH, (**C**) PSD-95, (**D**) GAP43, (**E**) GFAP, (**F**) MBP, (**G**) GLUR2, and (**H**) NMDAR1. No significant changes were observed in SYP, SPH, MBP, GLUR2 and NMDAR1 expression levels. Data are expressed as mean ± SEM, n = 6 animals per group, *p < 0.05, **p < 0.01. Full length blots are presented in Supplementary Fig. 3 (GAPDH was done on each blot after stripping the protein of interest on each corresponding blot).

We found a decrease in the expression levels of PSD-95 (t = 2.570; *p* = 0.027, Fig. 5C), GAP43 (t = 4.199; *p* = 0.001, Fig. 5D), and GFAP (t = 4.323; *p* = 0.001, Fig. 5E) in HA mice compared to SL mice. We did not observe significant changes in the expression levels of SYP (Fig. 5A), SPH (Fig. 5B), MBP (Fig. 5F), GLUR2 (Fig. 5G) and NMDAR1 (Fig. 5H) in HA mice compared to SL mice.

#### Brainstem

**Figure 6:**
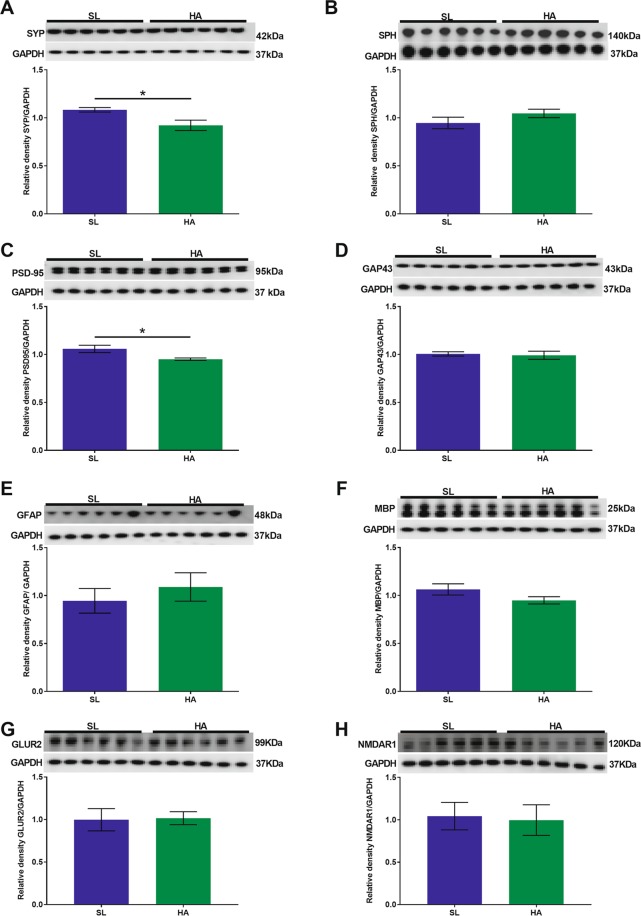
CEHA results in a significant decrease in the expression levels of SYP and PSD-95 in the brainstem. WB analysis of (**A**) SYP, (**B**) SPH, (**C**) PSD-95, (**D**) GAP43, (**E**) GFAP, (**F**) MBP, (**G**) GLUR2, and (H) NMDAR1. No significant changes were observed in SPH, GAP43, GFAP, MBP, GLUR2 and NMDAR1. Data are expressed as mean ± SEM, n = 6 animals per group, *p < 0.05. Full length blots are presented in Supplementary Fig. 4 (GAPDH was done on each blot after stripping the protein of interest on each corresponding blot).

We found a significant decrease in the expression levels of SYP (t = 2.746; *p* = 0.020, Fig. 6A) and PSD-95 (t = 2.704; *p* = 0.022, Fig. 6C) in HA mice compared to SL mice. We did not observe significant changes in the expression levels of SPH (Fig. 6B), GAP43 (Fig. 6D), GFAP (Fig. 6E), MBP (Fig. 6F), GLUR2 (Fig. 6G) and NMDAR1 (Fig. 6H) in HA mice compared to SL mice.

## Discussion

While CEHA has a global effect on the entire CNS, specific brain regions can be differentially affected based on their own levels of functional and molecular vulnerability^28^. Using our mouse model of CEHA we sought to investigate the possible effects induced by CEHA on the cognitive function as well as on the synaptic, astroglial and myelin protein expression in multiple and different brain regions. The major findings of our analyses were: a) chronic exposure to 5000 m for 12 weeks (CEHA) resulted in fear conditioning alterations which are considered signals of hippocampal dysfunction; b) CEHA leads to decreased levels of synaptic and astroglial related proteins across different regions of the brain.

A recent study by Kumari *et al*.^20^ found that rats exposed to 25,000 ft had significant deficits in cued and contextual fear acquisition, which peaked at seven days. In addition, other researchers have shown that rats kept at 6100 m for seven days had impairment in spatial memory^28^. Recently, we showed that mice exposed to HA for 3 weeks exhibited hippocampal dependent memory deficits that failed to recover over the course of a 12 week exposure^19^. Here, HA mice that were given a longer time to acclimate prior to testing (12 vs 3 weeks) showed similar deficits, supporting the hypothesis that cognitive impairments induced by CEHA are significant and persistent^19^. Moreover, the role and activation of different neuroanatomical structures of the brain in the fear conditioning have been reported. In a rat study it was reported that various neural networks are involved in olfactory fear conditioning using olfactory bulb stimulation induced by field potential signal (EFP), which acts as a marker of plasticity in the olfactory pathway^29^. Others have reported in both mice and rats that cerebellum also participates in fear learning and memory^30,31^. Besides that, the cerebellum also plays an important role in the classical fear conditioning in both mammals and non - mammals^32–35^. In another study, it has been observed that disruption of reticular-limbic central auditory pathway resulted in an impairment of noise-cued fear conditioning^36^. Pertaining to the role of each neuroanatomical structure has in the brain, we planned on studying: olfactory cortex, which has been shown to be the most ‘plastic’ region of the brain^37^; hippocampus, which is important for learning and memory and plays role in fear conditioning^38^; cerebellum, which, besides playing a role in balancing and coordination, also plays a role in learning and responding^39^; brainstem, which is the anatomical site to relay signals between the brain and spinal cord as well as for fundamental cardio-respiratory regulating centers^40–42^.

Interestingly, we did not observe differences in either synaptic density or morphology or synaptic, astroglial and myelin protein expression in the hippocampus. In contrast, however, Maiti *et al*.^28^ have shown decreases in dendritic branching of both apical and basal dendrites of pyramidal neurons in the CA1 and CA3 region of the hippocampus as well as in the cortex and striatum along with increased oxidative stress, DNA fragmentation, and apoptosis after seven days of HA exposure at 6100 m, but these abnormalities were restored after 21 days of exposure^28^. Nevertheless, this apparent contrast could be explained by the different experimental paradigm used and the different time points considered. Although cognitive function depends upon the level of altitude and duration of stay, it is possible that these changes in cognitive function may be due to an increased oxidative stress during the initial period of exposure and to a later adaption to that ascent. Moroever, recent human studies have shown that at HA the brain has adapted as there were no neurological symptoms left once returning to SL^43^. In our study, in the hippocampus, CEHA had no effect on the structure or morphology of synapses and there was no effect on the expression levels of SYP, SPH, and PSD-95. Given the function of these proteins, mainly but not only as scaffolding molecules, it is possible that the cognitive deficits observed in HA mice may be due to changes in downstream signaling proteins involved in neuroplasticity and synaptic function. In our recently published transcriptomic analyses data, we found that there was downregulation in neural activity-related RING finger protein (NARF) and metabotropic glutamate receptor (GRM3)^19^, which play, respectively, a crucial role in synaptic plasticity^44^ and learning and memory functions^45^.

The olfactory cortex is an anatomical region with major neuroplastic capacities and it is part of the anatomical circuit supporting the olfactory function^46–48^. Olfactory memory in terms of odor, learning and recognition plays an important role in the everyday lives of both animals and humans. Interestingly, we observed that CEHA leads indeed to a significant decrease in the expression levels of SYP and SPH in the olfactory cortex supporting the hypothesis that neuroplasticity mechanisms occur across the sensory synapses^49,50^. It is known that mice exposed to HA experienced significant initial weight loss and had a slower rate of weight gain compared to the weight gain of mice at SL^13^ (present study, data not shown). This weight loss and slow gain could be explained by either increased or decreased whole body metabolic rate at HA or by the olfactory function impairment as reduced sniffing capacities^51,52^, which may be associated to the reduced synaptic protein expression and consequent neuronal dysfunction in the olfactory cortex neurons. Moreover synaptic changes in the olfactory cortex of CEHA animals can be associated with alterations in the release of acetylcholine, a crucial neurotransmitter for memory function^53^.

The scientific literature shows that the cerebellum and brainstem are regions of the brain particualry vulnerable to HA exposure. In the brainstem, neonatal exposure to hypoxic-ischemic events results in oral-motor dysfunction, swallowing, and respiration abnormalities^54–59^ likely due to neurological deficits resulting from changes in monoamine and amino acid contents^54^. In addition, in the cerebellum, hypoxia delays the arborization of Purkinje cells in neonatal mice^60^. Humans who have experienced cerebellar degeneration have explicit alterations in motor refinement capability^61^. Intriguingly, our data show that CEHA leads to reduced expression of PSD-95, GAP43, and GFAP in the cerebellum. These results are actually of special importance given the putative role of the cerebellum in cognition in addition to its traditional role in motor learning and autonomic function. In fact, recently, long-term depression (LTD), has been found to occur in specific parts of the cerebellum thereby confirming and implicating its role not only in the motor or autonomic system, but in cognitive functions as well^58,62–66^. Given that PSD-95 is a stable scaffolding protein and helps in maintaining a balance between excitation and inhibition in the brain^67^, decrease of PSD-95 in the cerebellum and brainstem may influence synaptic strength and plasticity, which may have consequently an effect on cognition. GAP43, besides being a marker for new synapses, is associated with axonal growth, learning^68^ and neuroplasticity^69^ in granule cells of the cerebellum^70^ but not in Purkinje cells^71^, which may be one of the reasons for the observed decreased GAP43 expression thereby reducing cerebellar learning. It is known that SYP expression levels are associated with learning and memory changes and the levels of this protein may interfere with neuroplasticity processes. Given the role of SYP in vesicular transport in the presynaptic compartment, these changes in its expression resulting from CEHA may be an indicator of decreased pre-synaptic vesicles at the synapse which in turn relates to decrease in functional synapses^72,73^.

Previous studies have shown that astrocytes have diverse functions such as establishing direct contact with neurons^74–76^ and secreting soluble factors at the pre- and post-synaptic sites thus modulating the structure and function of both excitatory and inhibitory synapses^77–82^ leading to changes in synaptic transmission^83–85^. In regards to hypobaria-hypoxia, it has been shown that there is an increase in astrogliosis in the CA1 region of the hippocampus, as indicated by elevated expression of GFAP^86,87^. As further novelty, our findings reveal a decrease in GFAP expression in the cerebellum. It is intriguing that we observed these additional changes in the cerebellum, although at a different time point. A study by Dheer *et al*.^86^ studied CEHA in rats, where they observed activation of astrocytes in CA1 region of hippocampus at 7 and 14 days exposure; while Li *et al*.^87^, observed increased GFAP expression between 7–14 days in cortex and brainstem. The apparent discrepancy between our and their results could be due to multiple factors. One major factor is represented by the time duration, our mice in fact were exposed to a longer duration of HA, which is a chronic exposure, while their rats were exposed for a shorter period of time. Another difference is in the pressure of the hypobaric chamber, which was p = 282 mm/Hg (equivalent to 5.45 psi) for their experiments, and ~7.4 psi (equivalent to 382.69 mm/Hg) for our experiments. A further difference consists in their experimental animals, which were rats while ours were mice. All these factors could have contributed to the apparent discrepancy and it is possible then that the observed decrease in GFAP, along with the decrease in other synaptic proteins, are indeed signals of decreased synaptic activity in a more generalized context of neuroplasticity occurring in response to CEHA.

As for neurotransmitters, glutamate acts as an important mediator of synaptic transmission and plays a crucial role in diverse brain functions such as learning, memory, and cognition. Glutamate receptors such as AMPA receptor (AMPAR) - Glutamate receptor 2 (GluR2) subunit and NMDA (N-methylD-aspartate) - strongly influence receptor assembly, trafficking and are important for physiological information, processing, and long-term synaptic plasticity. Different studies have reported neural tissue injuries due to hypoxia that resulted in an increased accumulation of glutamate in the extracellular spaces^88^. For example, in three monthsold rats, exposed to 7600 m resulted in an increase in GLUR 2 expression at 3 days but at 7 and 14 days the expression was decreased^89^. In the same study, NMDA receptors were found to be decreased at day 3, while at 7 and 14 days the expression did not change substantially^89^. Similarly in our CEHA study, we did not observe any changes in the NMDAR1 and GLUR2 receptors in either SL and HA mice. This observation may support the fact that mice have been already adapted to CEHA exposure at the time point of our analyses and so it is not apparently affecting the levels of these receptors.

## Conclusion

In conclusion, the results from this study provide initial evidence that CEHA leads to alterations in the expression of various synaptic and astroglial proteins across different neuroanatomical regions (olfactory cortex, cerebellum and brainstem) in association with changes of the cognitive function. Our study provides the foundation for future research studies focusing on the understanding of many complex signaling pathways involved in synaptic plasticity and functions in response to CEHA as their role in neuroadaptive mechanisms. A better understanding of these mechanisms could also lead to the development of preventive tools against the deleterious neurological effects due to CEHA, especially for military and civilian populations required to work at HA for prolonged periods of time. The overview of CEHA and its role in synaptogenesis and plasticity in response to HA is a novel field of research which warrants further future investigation.

## Material and Methods

### Simulated HA exposure

Seven-week-old C57BL/6 male mice (n = 18) (Jackson Laboratories, Bar Harbor, ME, USA) were group housed on a reverse light cycle for at least one week prior to the experiment. HA mice were group housed in conventional cages inside a modified Vicker’s hypobaric chamber (Reimers System Inc., Lorton, VA, USA) for 12 weeks to operate under reduced pressures (~7.4 psi) using a vacuum pump (Welch Model 2585B or 2067B-01, Mount Prospect, IL, USA) as previously described^13,19^. To avoid acute effect, ascent to a simulated altitude of 5000 m (HA) proceeded at 200 m per minute and then returned to SL at the same rate. Environmental parameters (pressure, oxygen, carbon dioxide levels, temperature and relative humidity) inside of the chamber were continuously monitored using custom built sensors from CO_2_ Meter Inc. (Ormond Beach, FL, USA). The chamber was located within the animal vivarium to ensure consistent environmental parameters across exposure groups and conditions. The chamber altitude was monitored using a data logging digital manometer (AZ Instrument Corp., Taichung City, Taiwan). Cage maintenance was performed at SL at least once per week. All mice were monitored daily for signs of distress including failure to groom, and/or excessive weight loss.

### Behavioral tests for determination of memory

Mice were tested for cued and contextual fear memory as previously described^19,90,91^. Briefly, contextual memory deficits were evaluated at the end of 12 weeks of CEHA. For the initial training session, mice were acclimated to the testing room for five minutes prior to being placed individually in plexiglass chambers with a wire grid floor inside a dimly lit enclosure (Ugo Basile, Varese Italy). A black and white striped or checkered backdrop lining the walls of the enclosure was used to strengthen the context association. Mouse movements were recorded and analyzed for freezing behavior using ANY-maze software (Stoelting Co., Wood Dale, IL, USA). The training session consisted of a two minute acclimation period followed by a 30 second tone which ended with a two second, 0.5 mA foot shock. The tone-shock pairing was repeated one minute later and the mice were returned to their home cage after a final one minute period of monitoring for freezing. Mice were returned to their home environments (either at HA or SL) at the end of the test. Context-dependent memory formation was tested by returning the mice to identical chambers after 24 hours and monitoring freezing behavior in the absence of any additional tones or foot shocks for five minutes. After approximately 20 minutes back in their home cage environment, cue-dependent memory formation was tested by returning the mice to the testing chambers configured to have a novel environment (white walls, shock grid covered with plastic and vanilla odor). Three minutes after entering the novel environment, the original tone was played for three minutes followed by one minute of silence and freezing behavior was quantified for the entire period^19,90,91^. Based on the fear conditioning experiment, the mice were subdivided into different groups, n = 6 was used for western blot analyses, n = 6 was used for EM analyses, and the remaining mice were used for some other experiments not mentioned in this manuscript.

### Tissue preparation for electron microscopy

Mice were deeply anesthetized with intraperitoneal injection of 100 mg/kg Fatal Plus (Vortech Pharmaceuticals, Dearborn, Michigan, USA) and transcardially perfused with 1% paraformaldehyde in 0.1 m phosphate buffer followed by 4% paraformaldehyde and 0.125% glutaraldehyde. The brains were hemisected, postfixed overnight in 2% PFA and 2% glutaraldehyde at 4 °C and cut into 250 µm sections on a Vibratome (Leica VT1200S, Buffalo Grove, IL) and stored in 0.1 M cacodylate buffer. Following incubation in cacodylate buffer, brain samples were prepared for electron microscopy using epoxy resin embedding^92^. Briefly, samples were immersed in 2% osmium tetroxide for one hour, washed in cacodylate buffer and dehydrated using gradually increasing concentrations of ethanol. The samples were then immersed in Spurr epoxy resin (Electron Microscopy Sciences, Hatfield, PA), polymerized for 12 hours at 70 °C and cut into ribbons of thin serial sections (80 nm) using an Ultracut UC7 ultramicrotome (Leica Biosystems). Serial sections were carefully mounted onto formvar/carbon supported nickel slot grids, post-stained with lead citrate and urinal acetate (Leica EM AC20, Leica Biosystems).

### Quantitative analyses of synapse density

Grids were reviewed at 15,000x on a JEOL JEM-1011 transmission electron microscope (JEOL USA Inc., Peabody, MA). Nine sets of 5 serial sections in the stratum radiatum region of the CA1 were imaged using an AMT XR 50S-A digital camera. Images were imported into Adobe Photoshop (Adobe Photoshop CC 19.1.2) and adjusted for brightness and contrast prior to morphological analysis. Stereological unbiased quantitative analysis of synaptic density using the dissector method is routinely performed in the Dickstein’s laboratory^24–27^. Briefly, all axospinous synapses were identified within the first and last two images of each five-section serial set and counted if they were contained in the reference image but not in the corresponding look-up image. To increase sampling efficiency, the reference image and look-up image were then reversed; thus each animal included in the current study contributed synapse density data from a total of 18 dissector pairs. Perforated and non-perforated synapses were also quantified. For a synapse to be scored as perforated it had to display two or more separate post synaptic density (PSD) plates. Synaptic density was calculated as the total number of synapses that appeared in the reference or lookup images divided by the volume of the dissector used (14.8096 µm³). To measure PSD length and spine head diameter (the widest point of the spine head parallel to the PSD) each synapse identified in the lookup image was followed through the remainder of the five series. The longest PSD length and spine HD in those particular sections was identified and measured. For the smallest class of synapses that were only present in one serial section, measurements were taken in that section. For perforated synapses, the lengths of all PSD segments within a given section were summed and the total length was used in the statistical analyses.

### Tissue preparation for western blot analysis

On the day of euthanasia, mice were anesthetized with intraperitoneal injection of 100 mg/kg Fatal Plus (Vortech Pharmaceuticals, Dearborn, Michigan, USA). Perfusion was carried out for five minutes with 1X phosphate buffer saline (PBS) at pH 7.4. For western blot (WB) analysis, brains were dissected into four regions (olfactory cortex, cerebrum, cerebellum, and brainstem) immediately frozen in ice cold isopentane and stored at −80 °C until use. The frozen cerebrum was microdissected to isolate the hippocampus on the cryostat. Coronal sections were cut on a cryostat at 100 µm thick sections and then from each section the entire hippocampus was microdissected. We focused on specific synaptic proteins as indexes of possible pre-synaptic (SYP, SPH) and post-synaptic (PSD-95, SPH) changes as well as GAP43 alterations, a marker of neosynaptogenesis, triggered by or responding to CEHA^93^.

### Western blot analysis

Frozen tissue samples were homogenized in glass dounce homogenizers with ice cold lysis buffer (1 ml/100 mg tissue) containing the following: 50 mM Tris-HCl (pH = 8), 1% Igepal, 150 mM sodium chloride (NaCl), 1 mM ethylenediaminetetraacetic acid (EDTA), 1 mM phenylmethylsulfonyl fluoride (PMSF), 1 mM sodium fluoride (NaF), and 1:100 protease inhibitor cocktail (Sigma-Aldrich, P2714, St. Louis, MO, USA). Samples were centrifuged at 12,000 g for 20 minutes and supernatants collected, aliquoted and frozen at −80 °C. Total protein content from each brain region was determined using the Micro BCA assay (Thermo-Fisher Scientific, 23235, Waltham, MA, USA). For all analyses 10 µg of protein per sample were loaded on Novex Nupage 4–12% Bis-Tris Gels (Life Technologies, NP0329, Carlsbad, CA, USA) and were electrophoresed at 200 V for 30 minutes (SYP, PSD-95, GAP43, GFAP and MBP). Novex Nupage 4–12% Bis-Tris Gels (Life Technologies, NP0329, Carlsbad, CA, USA) and were electrophoresed at 200 V for 50 minutes (SPH). Gels were transferred to PVDF membranes using the iBlot2 dry transfer method (Life Technologies, IB21001, Carlsbad, CA, USA). Membranes were blocked in 5% milk in 1X Tris buffer saline 0.05% Tween 20 (TBST) for one hour at room temperature. The membranes were probed with primary antibodies diluted to the appropriate working concentrations in order to maintain a linear range of detectability across all antibodies.

We used the following primary antibodies: SYP (1:2000, Abcam, ab8049, Cambridge, MA, USA), SPH (1:1000, Cell signaling, 14136, Danvers, MA, USA), PSD-95 (1:1000, Neuromab,75-028, Davis, CA, USA), GAP43; 1:40000, Abcam, ab75810, Cambridge, MA, USA), GFAP (1:1000, Leica Biosystems, NCL-L-GFAP-GA5, Newcastle Upon Tyne, UK), MBP (1:2000, Abcam, ab62631, Cambridge, MA, USA), GLUR2; (1:2000; Millipore, MAB397, Burlington, USA), NMDAR1 (1:2000; Millipore, AB9864R, Burlington, USA) with 5% milk in 1X TBST followed by overnight incubation at 4 °C. Membranes were then rinsed three times for five minutes in 1X TBST. Appropriate HRP tagged secondary antibodies Goat anti-mouse (Abcam, ab97040, Cambridge, MA, USA); Goat anti-rabbit (Abcam, ab97080, Cambridge, MA, USA); Rabbit anti-chicken (Millipore-Sigma, AP162P, Billerica, MA, USA) were diluted 1:2000 with 5% milk in 1X TBS, and incubated on the membranes for one hour at room temperature. Membranes were rinsed three times for five minutes in 1X TBST and one time for five minutes in TBS. Membranes were incubated with chemiluminescent substrate (SuperSignal West Pico Chemiluminescent Substrate, Thermo-Fisher Scientific, 34577, Waltham, MA, USA) for one minute and imaged on the LiCor C-Digit Blot Scanner (LiCor Biosciences, Lincoln, NE, USA). All membranes were stripped one time with Restore Plus Stripping Buffer (Thermo-Fisher Scientific, 46430, Waltham, MA, USA), for ten minutes, rinsed with TBS and processed for immunoblotting as described above using GAPDH (1:40,000, Millipore-Sigma, AB2302, Billerica, MA, USA) as loading control. Densitometry was performed with NIH ImageJ software (2.0.0) with all protein signal intensities normalized to GAPDH signal intensity.

### Ethics approval

All animal procedures were carried out in accordance with National Institutes of Health and Institutional Animal Care guidelines and were approved by the Institutional Animal Care and Use Committee (IACUC) at the Uniformed Services University (USU), Bethesda, USA.

## Supplementary information

Dataset 1

## Data Availability

The datasets generated and/or analyzed during the current study are available from the corresponding author on reasonable request.
