# Supplementary material for: Chronic Exposure to High Altitude: Synaptic, Astroglial and Memory Changes
Source: Sci Rep. 2019 Nov 11;9:16406. doi: 10.1038/s41598-019-52563-1 (PMC6848138; doi:10.1038/s41598-019-52563-1)
Supplement: Supplementary file 1 — Dataset 1 [file 41598_2019_52563_MOESM1_ESM.pdf]

Chronic Exposure to High Altitude: Synaptic, Astroglial and Memory Changes

Rupali Sharma, PhD[1,2], Nathan P. Cramer, PhD[2,3], Bayley Perry, BSc[1,2], Zahra Adahman, MS[1,2], Erin K. Murphy, MS[1,2], Xiufen Xu, BS[2,3], Bernard J. Dardzinski, PhD[2,4], Zygmunt Galdzicki, PhD[2,3], Daniel P. Perl, MD[1,2] and Dara L. Dickstein, PhD[1,2], Diego Iacono, MD, PhD[1,2,5]

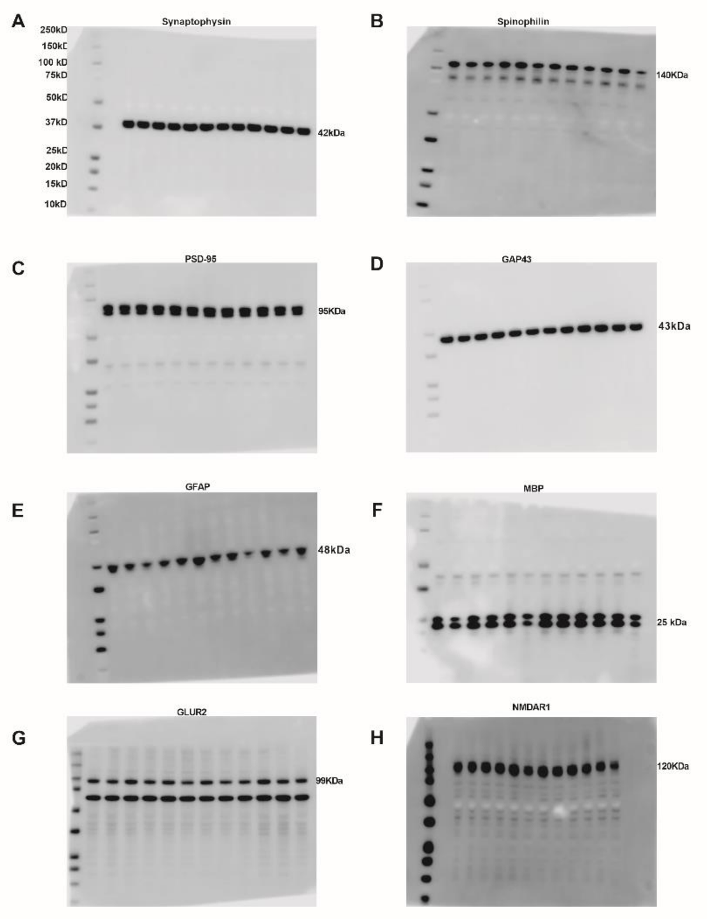

Supplementary figure 1: Full length blots of (A) Synaptophysin (B) Spinophilin (C) PSD-95 (D) GAP43 (E) GFAP (F) MBP (G) GLUR2 (H) NMDAR1 in the Olfactory cortex. GAPDH was done on each blot after stripping the protein of interest on each corresponding blot as shown individually in fig 3).

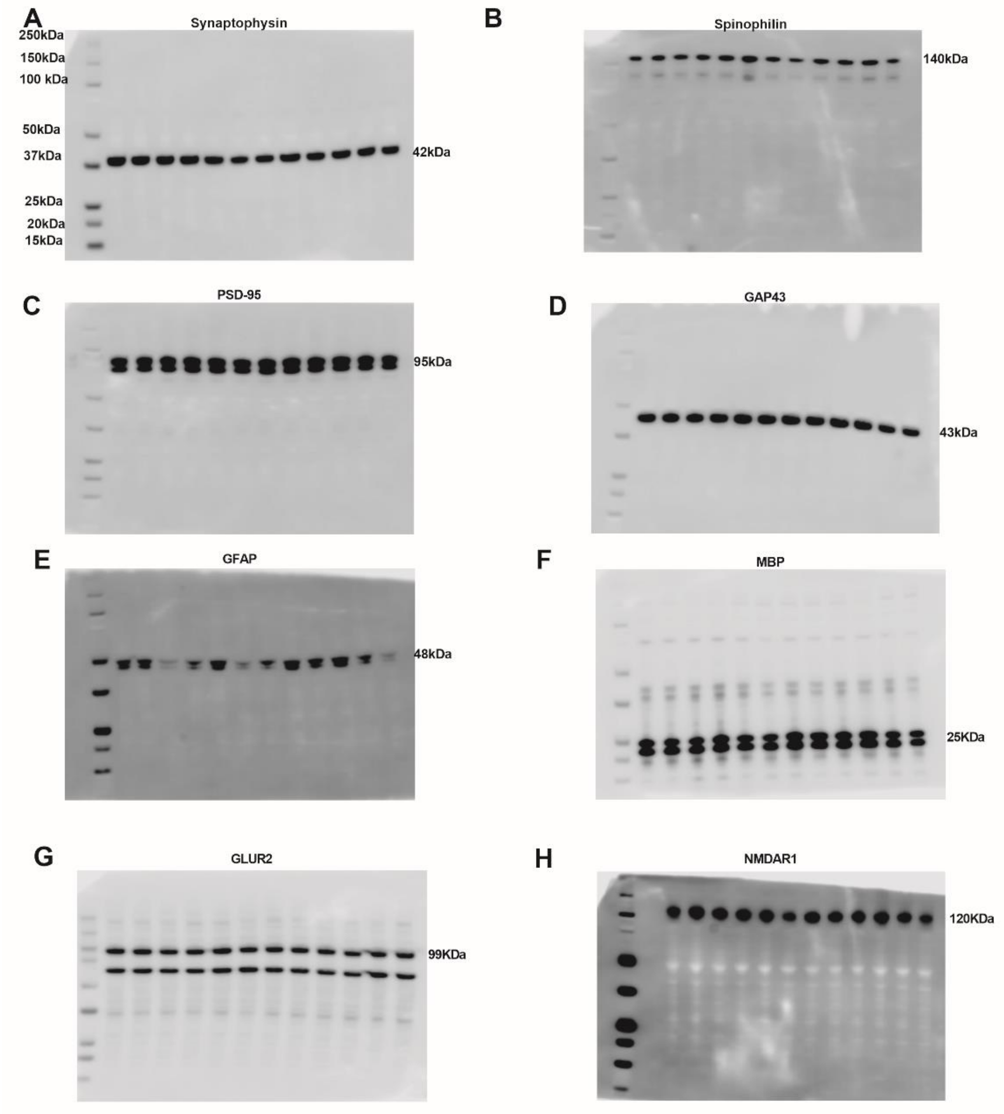

Supplementary figure 2: Full length blots of (A) Synaptophysin (B) Spinophilin (C) PSD-95 (D) GAP43 (E) GFAP (F) MBP (G) GLUR2 (H) NMDAR1 in the Hippocampus. GAPDH was done on each blot after stripping the protein of interest on each corresponding blot as shown individually in fig 4).

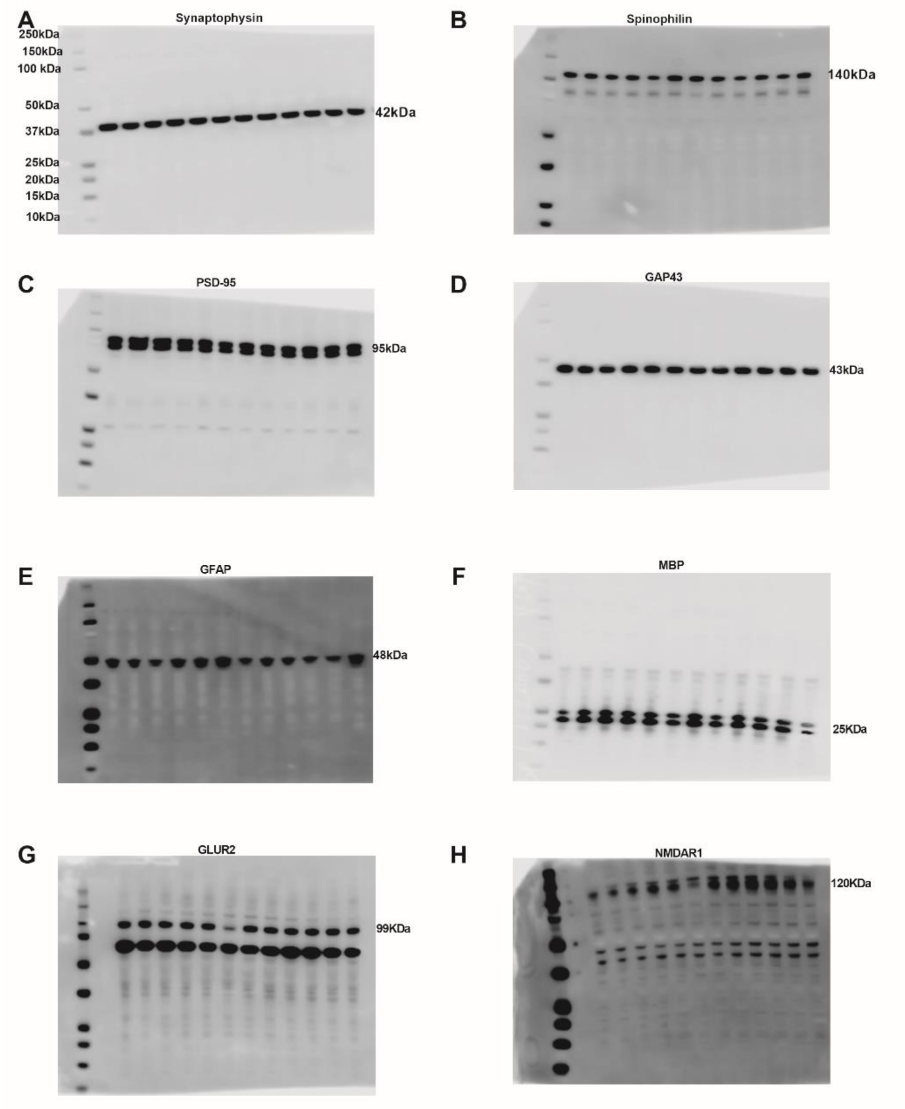

Supplementary figure 3: Full length blots of (A) Synaptophysin (B) Spinophilin (C) PSD-95 (D) GAP43 (E) GFAP (F) MBP (G) GLUR2 (H) NMDAR1 in the Cerebellum. GAPDH was done on each blot after stripping the protein of interest on each corresponding blot as shown individually in fig 5).

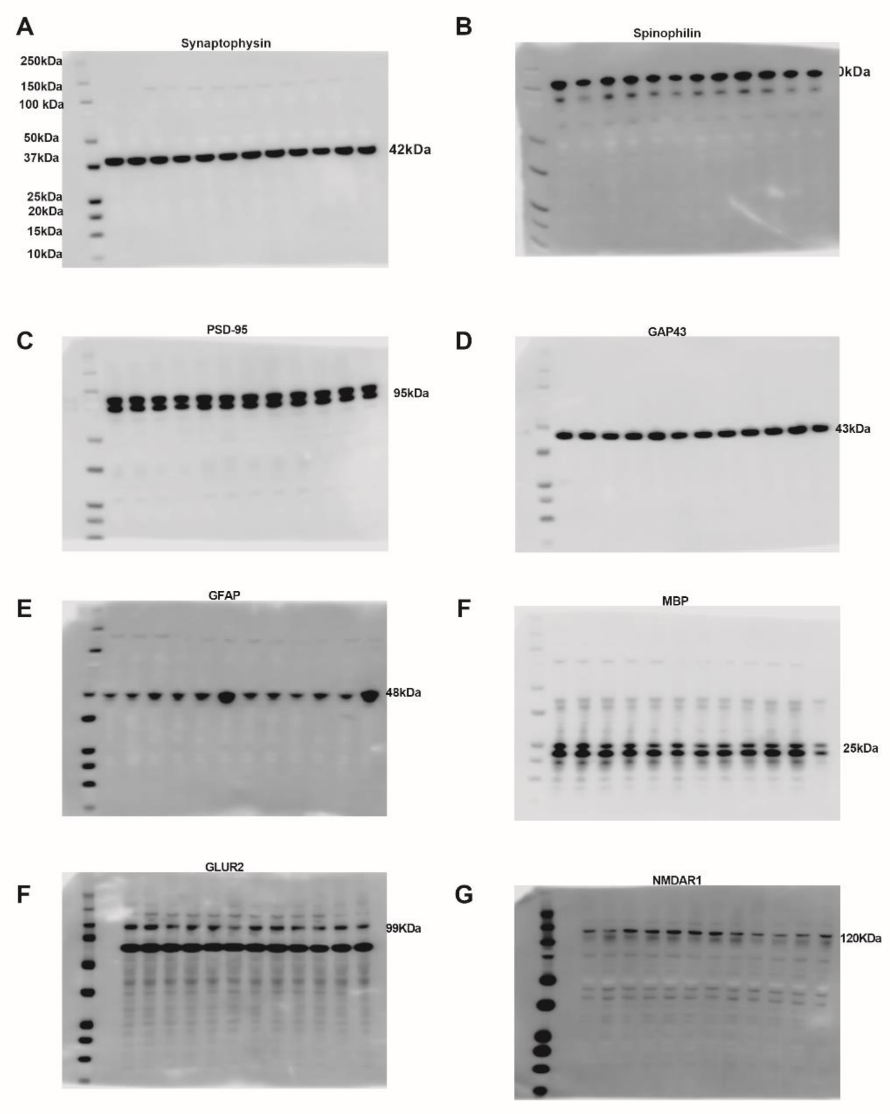

Supplementary figure 4: Full length blots of (A) Synaptophysin (B) Spinophilin (C) PSD-95 (D) GAP43 (E) GFAP (F) MBP (G) GLUR2 (H) NMDAR1 in the Brainstem. GAPDH was done on each blot after stripping the protein of interest on each corresponding blot as shown individually in fig 6).
